# Comparison of mechanical properties and host tissue response to OviTex™ and Strattice™ surgical meshes: author reply

**DOI:** 10.1007/s10029-023-02911-y

**Published:** 2023-10-19

**Authors:** J. Lombardi, E. Stec, M. Edwards, T. Connell, M. Sandor

**Affiliations:** Allergan Aesthetics, an AbbVie Company, 4 Millennium Way, Branchburg, NJ 08876 USA

**Keywords:** Biologic mesh, Hybrid mesh, Hernia, Acellular dermal matrix, Rodents, Primates

Dear Editor,

We thank Drs. Pacella and Nazerali for their feedback on the published article by Lombardi et al. “Comparison of Mechanical Properties and Host Tissue Response to OviTex™ and Strattice™ Surgical Meshes” (*Hernia* 2023;27:987–997) [1]. Drs. Pacella and Nazerali commented that the preclinical results were incomplete and unsubstantiated, and that the observed results were likely an artifact of cutting the meshes into 1 × 7 cm coupons. The in vitro collagenase digestion tensile test and rodent subcutaneous implant model used in this study were designed specifically to evaluate the material properties of surgical meshes under identical conditions, including trimming of both materials as permitted by the instructions for use. Although we agree that further mechanical analysis is warranted, this study focused on the strength retention of the materials as measured by maximum load (N/cm), the most critical parameter following both in vitro and in vivo enzymatic exposure. While we believe the data from these tests, previously used to evaluate biologic material properties and host response [2, 3], accurately reflect the device material characteristics under enzymatic conditions, we also agree that clinical insights should not be drawn from benchtop and rodent data alone.

Benchtop assays and rodent models have limitations, including potential xenogeneic responses and inability to fully assess mechanical fixation/force; therefore, these methods may not fully recapitulate the clinical scenario of abdominal wall repair (AWR). Given these limitations, we used a well-established non-human primate AWR model that was shown to have close immunologic homology to humans [4]. Observations from the non-human primate model corroborate the benchtop and rodent data in differentiating the enzymatic susceptibility of Strattice and OviTex, and further highlight the lesser role of the synthetic component in providing durability to OviTex. The results of these 3 models should be interpreted together for a complete preclinical comparison between Strattice and OviTex.

We concur that clinical studies are the gold standard for substantiating outcomes in clinical practice. Indeed, clinical studies support long-term durable outcomes with Strattice in patients undergoing complex ventral hernia repair [5, 6]. A retrospective cohort study that enrolled 725 patients who underwent AWR procedures, approximately half of whom received Strattice, reported cumulative hernia recurrence rates of 5%, 14%, and 18% with Strattice at 1, 3, and 5 years, respectively [5]. A prospective, observational, 14-year study (N = 362) reported recurrence rates of 3.7% after 1.7 years of follow-up [6]. Comparing outcomes across clinical studies is challenging due to differences in follow-up duration, meshes, and type/degree of patient complexity [5]; therefore, preclinical studies are useful to improve our understanding of biologic material properties and host response. Mesh selection remains complicated and ultimately requires informed decisions made by surgeons based on the individual needs of each patient.
